# Evaluation of molecular diagnostic approaches for the detection of BRAF p.V600E mutations in papillary thyroid cancer: Clinical implications

**DOI:** 10.1371/journal.pone.0179691

**Published:** 2017-06-21

**Authors:** Artur Kowalik, Aldona Kowalska, Agnieszka Walczyk, Renata Chodurska, Janusz Kopczyński, Magdalena Chrapek, Elżbieta Wypiórkiewicz, Małgorzata Chłopek, Liliana Pięciak, Danuta Gąsior-Perczak, Iwona Pałyga, Krzysztof Gruszczyński, Ewelina Nowak, Stanisław Góźdź

**Affiliations:** 1 Department of Molecular Diagnostics, Holycross Cancer Centre, Kielce, Poland; 2 Endocrinology Clinic, Holycross Cancer Centre, Kielce, Poland; 3 Department of Surgical Pathology, Holycross Cancer Centre, Kielce, Poland; 4 Department of Probability Theory and Statistics Institute of Mathematics Faculty of Mathematics and Natural Science, Jan Kochanowski University, Kielce, Poland; 5 Oncology Clinic, Holycross Cancer Centre, Kielce, Poland; 6 The Faculty of Health Sciences, Jan Kochanowski University, Kielce, Poland; CNR, ITALY

## Abstract

Differentiated papillary thyroid cancer (PTC) is the most common cancer of the endocrine system. PTC has a very good prognosis and a high 5 year survival rate; however, some patients are unresponsive to treatment, and their diagnosis eventually results in death. Recent efforts have focused on searching for prognostic and predictive factors that may enable treatment personalization and monitoring across the course of the disease. The presence of the BRAF mutation is considered to contribute to the risk of poor clinical course, according to American Thyroid Association (ATA) recommendations. The method used for genotyping can impact the predicted mutation frequency; however, ATA recommendations do not address this issue. We evaluated the molecular diagnostic (BRAF p.V600E mutation) results of 410 patients treated for PTC. We thoroughly analyzed the impact of three different *BRAF* mutation detection methods, Sanger Sequencing (Seq), allele-specific amplification PCR (ASA-PCR), and quantitative PCR (qPCR), on the frequency of mutation detection in 399 patients. Using Seq, we detected the *BRAF* mutation in 37% of patients; however, we were able to detect *BRAF* mutations in 57% and 60% of patients using the more sensitive ASA-PCR and qPCR technologies, respectively. Differences between methods were particularly marked in the thyroid papillary microcarcinoma group; BRAF p.V600E mutations were found in 37% of patients using Seq and 63% and 66% of patients using ASA-PCR and qPCR, respectively. We also evaluated how these different diagnostic methods were impacted by DNA quality. Applying methods with different sensitivities to the detection of BRAF p.V600E mutations may result in different results for the same patient; such data can influence stratification of patients into different risk groups, leading to alteration of treatment and follow-up schemes.

## Introduction

Differentiated thyroid cancer is the most common cancer of the endocrine system. In recent decades, the incidence of this type of cancer has increased 3-fold [1, 2]. In Poland, however, the incidence of this type of cancer has quadrupled (http://onkologia.org.pl/). The most common histological type of differentiated thyroid cancer, representing 80–85% of all cases, is papillary thyroid cancer (PTC) [3]. PTC has a very good prognosis and a high 5 year survival rate; however, some patients are unresponsive to treatment, and their diagnosis eventually results in death. Recent efforts have focused on searching for prognostic and predictive factors that may enable treatment personalization and monitoring across the course of the disease. Previously recognized PTC prognostic factors include tumor size, histological type, age, gender, the presence of distant or regional lymph node metastases, and incomplete tumor resection [4]. In terms of genetic susceptibility, the most important molecular variant is the BRAF p.V600E mutation, encoded by the c.1799T>A change in the *BRAF* gene, which causes the protein to become constitutively active. BRAF is part of the MAPK pathway, which blocks apoptosis and regulates proliferation and invasiveness [3]. Mutations in *BRAF* have been detected at different frequencies in different populations, with 36–67% of all PTC cases in the United States and Europe, and 90% in Korea, reported to carry mutations in this gene [3, 5–9]. The prognostic significance of *BRAF* mutations remains a topic of debate, and it has not been possible to unambiguously assess the clinical relevance of these mutations to the clinical progression of PTC [8–10]. Furthermore, the method used for genotyping can impact the detected mutation frequency. Depending on the technique applied, the frequency of mutations identified ranges from 37% for Sanger Sequencing (Seq), single-strand conformation polymorphism (SSCP), or mutant allele-specific PCR amplification (MASA); to 66% for quantitative PCR (qPCR); 68% for pyrosequencing; and 90% for peptide nucleic acid clamp real-time PCR [9, 11–14]. Using immunohistochemistry (IHC) to predict the presence of the p.V600E variant has yielded ambiguous results [15, 16]; however, a recently published mutation analysis by IHC revealed promising results, although further validation is required [17, 18]. Another factor that hinders the assessment of methodology for p.V600E mutation detection and its clinical significance is the clonality of tumor tissue in PTC, which necessitates the use of more sensitive methods, such as qPCR or pyrosequencing, rather than Seq [19–22].

In the latest recommendations of the American Thyroid Association (ATA), the presence of the BRAF p.V600E mutation is one of the factors taken into account in stratification for the risk of a poor clinical course in PTC; hence the results of screening for p.V600E have an impact on patient monitoring. Nevertheless, at present, the ATA recommendations do not take into account the methodology used to assess *BRAF* mutation status [23].

The goal of this investigation was to compare three genotyping methods, allele-specific amplification PCR (ASA-PCR), qPCR, and Seq, for detection of the BRAF p.V600E variant in 410 PTC cases. In addition, we analyzed the relationship between DNA quality, genotyping technique, and molecular diagnosis. We also assessed the impact of tumor tissue clonality, and calculated the mutation detection rate depending on the diagnostic method used.

## Materials and methods

### Ethics statement

All of the study procedures were approved by the Institutional Review Board at Holycross Chamber of Physicians in Kielce and performed according to the Declaration of Helsinki. All patients provided signed, informed consent before enrolling in the study.

### Patients

We collected formalin fixed paraffin embedded tissue blocks between January 2013 and June 2014 to analyze the *BRAF* mutation status for 410 patients treated for PTC at the Endocrinology Clinic of the Holycross Cancer Center (Kielce, Poland). Three hundred and sixty-one of the patients (88%) were women, and 49 (12%) were men. The mean age of the patients at the time of diagnosis was 50 years (SD = 12 years; range: 15–80 years).

The purpose of our analysis was to assess the impact of *BRAF* mutations on clinical course. Patients were divided into two groups: the first group included patients with papillary microcarcinoma (PTMiC; tumor diameter <10 mm) and without extrathyroidal extension; the second group included patients with papillary macrocarcinoma (PTMaC; tumor diameter >10 mm). We performed molecular studies using postoperative material.

### Molecular methods

We used three techniques (Seq, ASA-PCR, and real-time qPCR) to test for the presence of the p.V600E mutation in BRAF. The details of these techniques have previously been described in the literature [13, 15].

The pathologist marked the area containing PTC tumor cells on a hematoxylin- and eosin-stained slide. Then, the tumor tissue on matched unstained slides was deparaffinized and the pathologist-selected area was transferred to a tube for DNA isolation using the QIAamp DNA FFPE Tissue kit, according to the manufacturer’s instructions (Syngen, Wroclaw, Poland).

**Fig 1:**
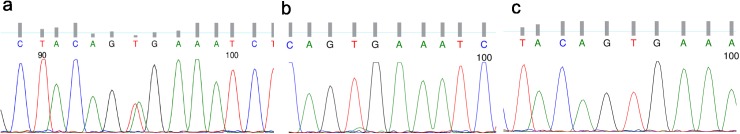
**Sequencing chromatograms representing three different types of results:** a) mutation *BRAF* c.1799T>A (p.V600E) present; b) ambiguous result; c) wild-type.

We amplified a 224 bp segment of *BRAF* exon 15 containing codon 600 using the following PCR primers: BRAFek15f (5′-TCATAATGCTTGCTCTGATAGGA-3′) and BRAFek15r (5′-GGCCAAAAATTTAATCAGTGGA-3′). After purification of the PCR products, sequencing was performed using a BigDye Terminator v1.1 Cycle Sequencing kit (Life Technologies, Warsaw, Poland) and an ABI 3130 Automatic Capillary DNA Sequencer (Applied Biosystems, USA). We discriminated among three different types of result: mutation present, mutation absent (wild-type; WT), and ambiguous (sequencing results not clearly interpretable; Fig 1).

**Fig 2:**
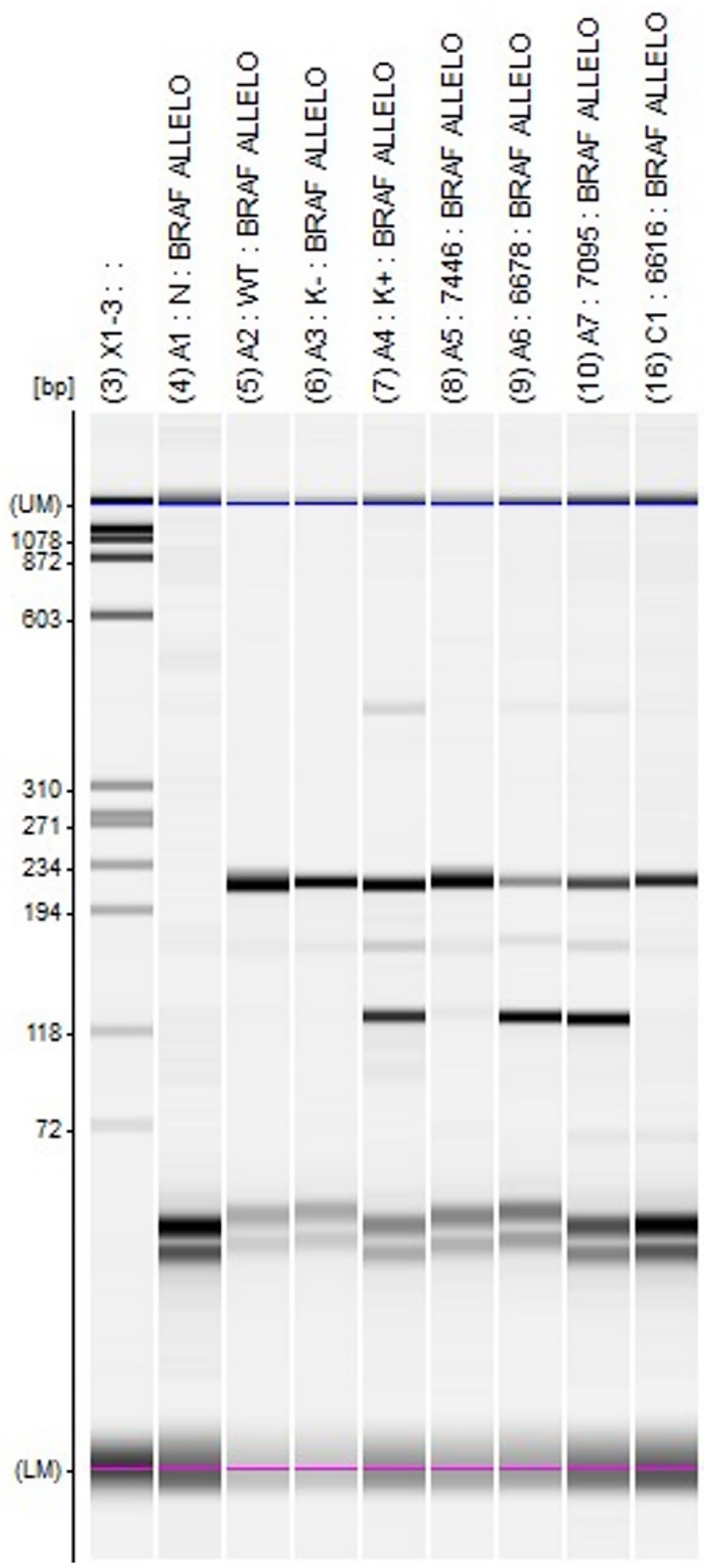
*BRAF* c.1799T>A (p.V600E) mutation genotyping results using the ASA-PCR method visualized by MultiNA chip electrophoresis (Shimadzu, Japan). X1, molecular weight marker; A1, no template control; A2, negative control (sample without the BRAF p.V600E mutation; WT); A3, negative control sample without mutation (K-); A4, control sample with BRAF p.V600E mutation (K+); A5–A7 and C1, tested samples. 224pz, reaction control band; 126pz, band indicating the presence of the p.V600E mutation.

Allele-specific PCR uses three types of primers. Common forward and reverse primers amplify a DNA fragment with the mutation in the center. The third primer is typically a forward primer that perfectly matches the mutated allele and amplifies it efficiently. By contrast, the mismatched allele is poorly amplified, if at all, by the mutation-specific primer. Unlike qPCR, ASA-PCR is an endpoint reaction. We performed allele-specific PCR using the BRAFek15f and BRAFek15r primers noted above to generate a 224 bp control band and a p.V600E mutation-specific primer (BRAFek15mutA 5′-GGTGATTTTGGTCTAGCTACAGA-3′) together with BRAF15r to amplify a 126 bp mutant fragment. We included positive and negative controls in each experiment. When the control band was not visible (possibly due to DNA degradation), the results were uninterpretable. ASA-PCR products were visualized using MultiNA automatic chip based electrophoresis (Shimadzu, Kyoto, Japan) (Fig 2).

**Fig 3:**
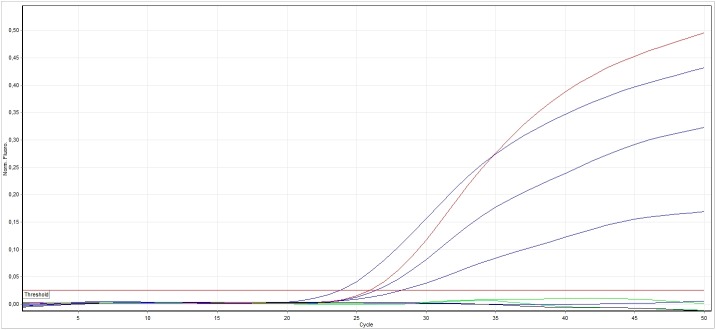
Results of genotyping the *BRAF* c.1799T>A (p.V600E) mutation using qPCR. Black, no template control sample; green, control sample without the BRAF p.V600E mutation; red, control sample with the BRAF p.V600E mutation; blue, test samples.

We performed a qPCR assay targeting a 68 bp region of *BRAF* exon 15 using Rotor-Gene Q (Qiagen, Syngen-Biotech, Poland) with the primers, forward 5′-agacctcacagtaaaaataggtgattttgg-3′ and reverse 5′-gatgggacccactccatcg-3′, and *BRAF* mutant-specific (6FAM-CTACAGAGAAATC-MG-BNFQ) and *BRAF* WT allele-specific (VIC-CTACAGTGAAATC-MGB-NFQ) probes (Fig 3).

### Statistical analysis

McNemar’s test was used to compare paired proportions. Chi-squared test with Yates’ correction was used to compare independent proportions. Two-tailed *p* values <0.05 were considered statistically significant. Bonferroni correction was applied to correct for multiple comparisons. All computations were performed using the statistical package, *R*, version 3.1.2 (Vienna, Austria) [24].

## Results

In January 2010, we started molecular diagnostics of the *BRAF* mutation p.V600E in PTC using the Seq method. Subsequently, in early 2012, due to reports in the literature of a higher incidence of this mutation in PTC, we complemented our diagnostic algorithm with the more sensitive method, ASA-PCR, which helps to resolve the ambiguous results that can be produced by Seq. In 2013, for automation and to increase the sensitivity of BRAF p.V600E mutation detection, a third method, qPCR, was added.

Four hundred and ten cases were selected that had been screened for the p.V600E mutation between 2013 and 2014, and the results were subjected to detailed statistical analysis. We planned to test the detection of the p.V600E variant using each of the three methods; however, no tests were carried out in eight cases (six PTMiC and two PTMaC) due to a lack of DNA. In three cases (two PTMiC and one PTMaC), there was insufficient DNA material for the qPCR examination. After excluding these 11 cases, 399 cases (206 PTMaC and 193 PTMiC) were subjected to further analysis (Tables 1 and 2).

**Table 1:** Genotyping results depending on the diagnostic method used (S, Seq; A, ASA-PCR; q, qPCR) in the entire cohort of 399 cases.

	Method			p value*		
	S	A	q	S vs. A	S vs. q	A vs. q
**Failed reaction**	36 (9%)	32 (8%)	5 (1%)	0.58	<0.0001	<0.0001
**p.V600E mutation**	149 (37%)	229 (57%)	238 (60%)	<0.0001	<0.0001	0.03
**WT**	186 (47%)	138 (35%)	156 (39%)	<0.0001	0.001	0.0009
**Detected mutation other than p.V600E**	17 (4%)	−	−	−	−	−
**Ambiguous result**	11 (3%)	−	−	−	−	−

*Generated using McNemar’s test.

**Table 2:** Genotyping results according to tumor size (PTMiC or PTMaC) and diagnostic method (S, Seq; A, ASA-PCR; q, qPCR).

		Method			*p* value*		
		S	A	q	S vs. A	S vs. q	A vs. q
**p.V600E**	**PTMaC > 10 mm (n = 206)**	78 (38%)	108 (52%)	110 (53%)	<0.00001	<0.00001	0.683
	**PTMiC ≤ 10 mm (n = 193)**	71 (37%)	121 (63%)	128 (66%)	<0.00001	<0.00001	0.023
	*p* value**	0.91	0.030	0.011			
**WT**	**PTMaC > 10 mm (n = 206)**	98 (47%)	84 (41%)	94 (46%)	0.026	0.627	0.016
	**PTMiC ≤ 10 mm (n = 193)**	88 (46%)	54 (28%)	62 (32%)	<0.00001	0.0001	0.043
	*p* value**	0.768	0.01	0.008			
**Failed reaction**	**PTMaC > 10 mm (n = 206)**	14 (7%)	14 (7%)	2 (1%)	1.00	0.003	0.001
	**PTMiC ≤ 10 mm (n = 193)**	22 (11%)	18 (9%)	3 (2%)	0.453	<0.00001	0.0003
	*p* value**	0.15	0.456	0.942			

*Generated using McNemar’s test.

**Test of the proportion of independent samples (PTMaC vs. PTMiC).

Analysis of the entire data set (n = 399 cases) revealed that the detected mutation rate was dependent on the analysis method used. Seq detected 37% of mutations; however, the use of ASA-PCR and qPCR increased the mutation rate to 57% and 60%, respectively (*p* < 0.0001; Table 1). After Bonferroni correction for multiple testing, the p.V600E mutation rate only differed significantly between Seq and ASA-PCR and Seq and qPCR. The WT genotype was detected most frequently using Seq (47%). The difference in the incidence of the WT genotype was statistically significant between Seq and ASA-PCR (*p* < 0.0001), Seq and qPCR (*p* < 0.001), and ASA-PCR and qPCR (*p* < 0.0009) (Table 1). The frequent detection of the absence of the p.V600E mutation by qPCR (39%) compared with ASA-PCR (35%) was due to the fact that qPCR found no p.V600E mutations in the majority of cases where there was a failed reaction for the ASA method. The frequency of failed reactions in the entire study group (n = 399 cases) differed significantly between Seq and qPCR (*p* < 0.0001) and between ASA-PCR and qPCR (*p* < 0.0001) (Table 1).

Among the 11 ambiguous results generated by the Seq method, 10 were identified as having p.V600E mutations by both ASA-PCR and qPCR (Table 1).

Also, for both tumor types (PTMaC and PTMiC) there was a trend toward more frequent p.V600E mutation detection with the ASA-PCR and qPCR methods than the Seq approach (Table 2). The greatest disparity in the mutation detection rate among the methods was for PTMiC; the qPCR method detected nearly twice the number of cases than Seq (*p* < 0.0001; Table 2). Using Seq, p.V600E mutations were detected in PTMiC and PTMaC at almost identical frequencies (37% and 38%, respectively). By contrast, depending on the method used (ASA-PCR or qPCR), the variant was more frequently detected in PTMiC (63% and 66%, respectively; *p* = 0.03) than PTMaC (52% and 53%, respectively; *p* = 0.011). For both tumor groups, the frequency of the WT genotype differed significantly based on the method used (PTMiC: Seq vs. ASA-PCR, *p* < 0.00001; Seq vs. qPCR, *p* < 0.0001; ASA vs. qPCR, *p* = 0.043; PTMaC: Seq vs. ASA-PCR, *p* = 0.026 and ASA vs. qPCR, *p* = 0.016). Comparing between tumor types, the frequency of the WT genotype differed significantly between PTMiC and PTMaC using ASA-PCR (*p* = 0.01) and qPCR (*p* = 0.008), but not when Seq was used. In addition, samples failed genotyping more often when Seq or ASA-PCR was used rather than qPCR (*p* < 0.0001), which affected the number of WT results (Table 2).

Mutations other than p.V600E were more frequently detected using Seq in PTMaC than PTMiC (6% vs. 2%); however, this difference was not statistically significant (*p* = 0.18). Similarly, ambiguous results occurred more frequently in PTMiC than PTMaC (4% vs. 2%); again, the difference was not statistically significant (*p* = 0.47).

## Discussion

PTC is the most commonly diagnosed cancer of the thyroid gland. It is characterized by a generally mild course, which is reflected in a very high 5 year survival rate (>95%). Unfortunately, some patients die due to PTC, despite seemingly adequate treatment; therefore, it remains critically important to discover biomarkers that can stratify patients into groups based on their predicted prognoses [10, 25–27]. One extensively studied molecular marker is the p.V600E mutation in the *BRAF* gene [9]. According to the current ATA 2015 recommendations (published in 2016), the presence of the BRAF p.V600E mutation does not alter the way patients are treated; however, together with other clinical factors, it is included in risk stratification for poor clinical course. In PTMiC, the presence of the BRAF p.V600E mutation is not useful for estimation of recurrence risk. However, for PTMaC, the risk of recurrence of structural disease in the presence of this mutation is estimated at approximately 10%, while in the absence of the mutation the risk is about -5% [23].

In this study, we compared the results of three methods for genotyping the p.V600E mutation in 399 PTC patients. Large differences in the frequency of this mutation, depending on the population studied and the method of detection used, have been noted in the literature [9, 28]. In the current comparative study, we found that p.V600E was detected in 37% of PTC cases using the Seq method; however, when we used more sensitive techniques (ASA-PCR and qPCR), mutation frequencies increased to 57% and 60%, respectively. Prior to this study, mutations in the Polish population had been analyzed by two research groups [29, 30]. In one study, mutations were detected in 12/25 PTC cases (48%) using SSCP and real-time quantitative ASA-PCR. We attribute these comparatively lower mutation frequencies to the interpretation of the genotyping test results, although the differences may also be due to the small size of the study group in the previous report [29]. In the second Polish study, the authors performed a molecular analysis of 88 PTC cases using the Seq method [30] and reported a mutation frequency of 43%, which is higher than that found in the current study using the same method; however, Czarniecka et al. did not consider ambiguous results, indicating that their method of results interpretation differed from ours. The Seq results generated in the current study were very similar to those from American and European studies that have used this method [7, 9]. Additionally, our results obtained using more sensitive methods (qPCR and ASA-PCR) are similar to those obtained by other groups. For example, Huang et al. detected *BRAF* mutations in 55% of subjects using amplification-refractory mutation system PCR, compared with our result of 57% [31]. In the articles discussed above, the authors focused on determination of the limit of sensitivity of the techniques applied using dilutions of DNA samples carrying the c.1799T>A mutation derived from cell lines [11, 14, 31]. The specificity of p.V600E mutation detection by PCR based methods was assessed by inclusion of follicular thyroid carcinoma, follicular adenoma, and normal thyroid among the test samples, in which p.V600E mutations were not detected [11, 31].

Guerra et al. reported p.V600E mutation rates of 36.9% and 53.6%, detected using Seq and pyrosequencing, respectively [21]. Other researchers detected the p.V600E mutation in 73% of an American population, using qPCR based methods [32]. These results confirm the trend observed in this study, where a higher mutation frequency was noted when more sensitive methods were used. By contrast, the extremely high mutation rates (66–90%) observed in the Korean population cannot be explained only by the use of a more sensitive genotyping method. In particular, the p.V600E mutation was detected in 68% of cases by pyrosequencing [12, 14]; however, it should be noted that this mutation was detected at a frequency of 53.6–56.9% by an Italian group using pyrosequencing, with a cut-off point of 5%, compared with the 3.2% value used in the Korean studies [12, 19, 21]. Ethnic, geographical, and environmental factors, as well as diet (consumption of large amounts of iodine in the form of seaweed), may have a substantial impact on the frequency of the BRAF p.V600E mutation in Eastern populations [12, 14]. For example, Chinese researchers reported an overall BRAF p.V600E mutation rate of 62% using the Seq method [33]. The authors observed a relationship between the dietary supply of iodine and the frequency of mutations, with mutation frequencies of 69% in regions with diets rich in iodine and only 53% in regions with iodine-deficient diets [33]. Additionally, only one of the studies described above used fresh tumor tissue samples alone [29]. Application of FFPE material in molecular studies is justified due to simpler established workflows for sample processing in pathology departments. In addition, collection of frozen material (often tumors are <10 mm in diameter) requires a great deal of effort, in terms of organization and equipment. Guerra et al. [21], who used frozen and FFPE samples, did not report any differences between the genotyping results from the two sample types. Furthermore, in all the above-mentioned studies, the frequencies of detection of the p.V600E mutation were consistent with the results obtained in this study, according to the methods used.

In the current study, using more sensitive methods (qPCR and ASA-PCR), approximately 15% of cases that were classified by Seq as having the WT allele actually proved to be carriers of the mutation (Table 1). Possible explanations for this finding include heterogeneity of the tumor tissue and the amount of normal cell contamination of tumor cells in the material used for genotyping. In our cohort, all but one of the samples had more than 10% of tumor cells in the tissue sample. The percentage of tumor cells in the studied cases was in the range 10–95% (mean 58%, median 60%). We did not find any significant differences between mutation calling when we compared the results from cases with 10–20% tumor cells with those with 25–95% analyzed by the same molecular test (Seq, ASA, qPCR). As the DNA extraction step was preceded by histopathological evaluation of the tumor cell content of the sample, the differences in detection rates may correspond to the heterogeneity of the tumor cell populations. In the context of tumor cell heterogeneity, it is preferable to use the most sensitive method for BRAF p.V600E mutation detection. In our hands, qPCR was the most sensitive technique for BRAF p.V600E mutation detection, because it could detect the mutation even when samples were very heterogeneous. Application of sensitive genetic methods (qPCR, next-generation sequencing (NGS), MassARRAY, or pyrosequencing) and IHC has demonstrated that there is considerable heterogeneity in the presence of the BRAF p.V600E mutation in populations of PTC tumor cells [20, 21, 34].

Our results indicate that Seq and ASA-PCR are equally dependent on the quality of the tumor sample. In the current study, the genotyping failure rate ranged from 1% to 9% (Table 1) depending on the diagnostic method used. Similar results were obtained by Ilie et al. (2–9%) [18]. Our results revealed that the qPCR method is less sensitive to degradation compared with Seq and ASA-PCR, probably due to the length of the fragments amplified in each method; for qPCR, the amplified fragment was only 68 bp, compared with 224 bp for both Seq and ASA-PCR. The results presented by Ilie et al., who used Seq, pyrosequencing, and SNaPshot techniques, were similar [18]. This phenomenon is associated with DNA modification and degradation during formalin fixation and paraffin embedding [35, 36].

Based on our results, the incidence of the BRAF p.V600E mutation in PTC in the patients tested may be similar to that indicated by the results of qPCR genotyping (i.e., approximately 60%) [37]. The qPCR method has been validated in a multicenter and multinational study [15] and is currently the method of choice in our department for *BRAF* c.1799T>A genotyping. The drawback of this approach is its inability to analyze other mutations; however, such mutations are rare (3% in the current study), and it is unclear whether they are clinically significant [38]. Furthermore, to date, there are no inhibitors available to effectively block these mutations. We cannot exclude other mutations in the p.V600 codon or nearby that were not detected by qPCR because of lack of complementarity of the TaqMan probe. Problems related to tumor clonality and the presence of different mutations in the *BRAF* gene may be solved by NGS, which is a robust technology that could, in the future, function as a substitute for other techniques (qPCR, Seq, pyrosequencing) because of its superior sensitivity and ability to comprehensively assess mutation composition in tumor tissue in a single analysis. NGS may also yield information about the proportion of cells carrying a given mutation, which could help to infer the clonal composition of the tumor tissue. Furthermore, NGS should be invaluable for targeted therapy stratification. Assessment of more genes could better refine diagnoses and may be helpful for prognostication purposes [39].

## Conclusions

Our analysis revealed that the BRAF p.V600E mutation rate detected in PTC cases depends on the sensitivity of the genotyping method used; in the current study, the frequency ranged from 37% (Seq) to 60% (qPCR). Additionally, qPCR proved to be the least dependent on the quality of the DNA used for the genetic diagnostics. Accordingly, the method of choice for the analysis of the BRAF p.V600E mutation should be one with sensitivity in the order of 1–5%, that is robust to DNA degradation (e.g., qPCR or pyrosequencing).

The use of different methods to detect the *BRAF* mutation can yield significantly different results for the same patient, and this is particularly evident for microcarcinomas. The presence of the *BRAF* mutation is taken into account in ATA risk stratification for structural disease recurrence, and impacts the intensity of further treatment and follow-up for patients. Therefore, it is necessary to clearly specify which method is appropriate for BRAF p.V600E mutation testing and to ensure that the results have predictive value [23].
